# Inflammatory cascades mediate synapse elimination in spinal cord compression

**DOI:** 10.1186/1742-2094-11-40

**Published:** 2014-03-04

**Authors:** Morito Takano, Soya Kawabata, Yuji Komaki, Shinsuke Shibata, Keigo Hikishima, Yoshiaki Toyama, Hideyuki Okano, Masaya Nakamura

**Affiliations:** 1 Department of Orthopedic Surgery, Keio University School of Medicine, 35 Shinanomachi, Shinjuku-ku, Tokyo 160-8582, Japan; 2 Department of Physiology, Keio University School of Medicine, 35 Shinanomachi, Shinjuku-ku, Tokyo 160-8582, Japan; 3 Central Institute for Experimental Animals, 3-25-12 Tonomachi, Kawasakiku, Kawasaki, Kanagawa 210-0821, Japan

**Keywords:** cervical compressive myelopathy, tip-toe walking Yoshimura mice, complement activation classical pathway, synapse elimination

## Abstract

**Background:**

Cervical compressive myelopathy (CCM) is caused by chronic spinal cord compression due to spondylosis, a degenerative disc disease, and ossification of the ligaments. Tip-toe walking Yoshimura (*twy*) mice are reported to be an ideal animal model for CCM-related neuronal dysfunction, because they develop spontaneous spinal cord compression without any artificial manipulation. Previous histological studies showed that neurons are lost due to apoptosis in CCM, but the mechanism underlying this neurodegeneration was not fully elucidated. The purpose of this study was to investigate the pathophysiology of CCM by evaluating the global gene expression of the compressed spinal cord and comparing the transcriptome analysis with the physical and histological findings in *twy* mice.

**Methods:**

Twenty-week-old *twy* mice were divided into two groups according to the magnetic resonance imaging (MRI) findings: a severe compression (S) group and a mild compression (M) group. The transcriptome was analyzed by microarray and RT-PCR. The cellular pathophysiology was examined by immunohistological analysis and immuno-electron microscopy. Motor function was assessed by Rotarod treadmill latency and stride-length tests.

**Results:**

Severe cervical calcification caused spinal canal stenosis and low functional capacity in *twy* mice. The microarray analysis revealed 215 genes that showed significantly different expression levels between the S and the M groups. Pathway analysis revealed that genes expressed at higher levels in the S group were enriched for terms related to the regulation of inflammation in the compressed spinal cord. M1 macrophage-dominant inflammation was present in the S group, and cysteine-rich protein 61 (Cyr61), an inducer of M1 macrophages, was markedly upregulated in these spinal cords. Furthermore, C1q, which initiates the classical complement cascade, was more upregulated in the S group than in the M group. The confocal and electron microscopy observations indicated that classically activated microglia/macrophages had migrated to the compressed spinal cord and eliminated synaptic terminals.

**Conclusions:**

We revealed the detailed pathophysiology of the inflammatory response in an animal model of chronic spinal cord compression. Our findings suggest that complement-mediated synapse elimination is a central mechanism underlying the neurodegeneration in CCM.

## Background

Cervical compressive myelopathy (CCM) is caused by chronic spinal cord compression due to spondylosis, a degenerative disease of the cervical discs, and ossification of the posterior longitudinal ligaments or yellow ligaments [1,2]. The symptoms appear mainly in the elderly, and include slowly progressive clumsiness and paresthesia in the hands, gait disturbance, and tetraplegia. Human histological studies revealed degeneration of the anterior horns, cavity formation, and demyelination in the severely compressed spinal cord [3,4]. Reports on the surgical outcomes of these patients demonstrate that increased spinal cord stenosis is associated with a worse postoperative recovery [5,6]. Although severe spinal cord compression is known to cause irreversible neurological damage, it is unclear how these pathological changes occur.

Tip-toe walking Yoshimura (*twy*) mice, which develop progressive spinal cord dysfunction secondary to extradural calcified deposits at the C2/3 ligaments, are reported to be a good in vivo model for the pathological changes related to CCM [7-9]. Because this mouse develops spinal cord compression spontaneously, there are individual differences in the severity of spinal cord compression [10]. Previous histological studies have shown that neurons are lost due to apoptosis in *twy* mice [11-13], but the exact mechanism of the neurodegeneration has not been fully elucidated. The purpose of this study was to investigate the pathophysiology of CCM by evaluating the global gene expression of the compressed spinal cord and comparing the transcriptome analysis with physical and histological findings in *twy* mice.

## Methods

### Animal model

The *twy* mice were obtained from a breeding colony of the Central Institute for Experimental Animals (Kawasaki, Japan). The mutant *twy* mice were maintained by brother-sister matings of heterozygotes at the Central Research Institute [11,14]. The *twy* mice harbor an autosomal recessive mutation in the nucleotide pyrophosphatase (*NPPS*) gene [7]. The mice were housed in groups under a 12-hour light/dark cycle, with access to food and water ad libitum. All experiments were performed in accordance with the Guidelines for the Care and Use of Laboratory Animals of Keio University School of Medicine and the Central Institute for Experimental Animals.

### Magnetic resonance imaging

Magnetic resonance imaging (MRI) was performed on the mice at 6, 15, and 20 weeks of age using a 7.0-Tesla magnet (BioSpec 70/16; Bruker BioSpin, Ettlingen, Germany) with a cryogenic quadrature RF surface probe (CryoProbe; Bruker BioSpin AG, Fällanden, Switzerland) to improve the sensitivity [15-18]. The MRI was performed under general anesthesia induced by the intramuscular injection of ketamine (50 mg/kg; Sankyo, Tokyo, Japan) and xylazine (5 mg/kg; Bayer, Leverkusen, Germany) and maintained by isoflurane (Foren; Abbott, Tokyo, Japan). The animal’s pulse, arterial oxygen saturation, and rectal temperature were monitored during the MRI. The scanning parameters were as follows: Saggital T2-weighted images (RARE, eTE/TR: 37.5/2000 ms), axial T2-weighted images (RARE, eTE/TR: 21.5 ms/1200 ms). To examine the extent of spinal cord compression due to extradural calcified deposits, the transverse areas of the calcification and spinal canal were measured on the axial T2-weighted images of the *twy* mice, and the canal stenosis ratio was calculated as reported previously [10].

### Behavioral analyses

The motor function of 20-week-old *twy* mice was evaluated using a Rotarod treadmill apparatus (Muromachi Kikai Co., Ltd., Tokyo, Japan) and the DigiGait Image Analysis System (Mouse Specifiics, Quincy, MA, USA). In the Rotarod treadmill test, the time (latency) that each mouse spent on the rod as it rotated at 10 rpm in a 2-min session, was monitored [19]. Three trials were conducted, and the average number of seconds was recorded. In the footprint analysis using the Digigait system, the stride length of the fore and hindlimb was measured as long as the *twy* mouse could walk with consistent weight-supported forelimb steps, on a treadmill set at a speed of 8 cm/s.

### Gene expression analysis

After the in vivo MRI analysis, the *twy* mice were anesthetized and transcardially perfused with heparinized saline (5 U/ml). Dissected segments of the cervical spinal cords were rapidly frozen and placed in TRIzol (Invitrogen, CA, USA). The total RNA was isolated using an RNeasy Mini Kit (Qiagen, Hilgen, Germany) according to the manufacturer’s instructions. For the microarray analysis, Cyanine-3 (Cy3)-labeled cRNA was prepared from 100 ng of RNA using the One-Color Low RNA Input Liner Amplification kit (Agilent, CA, USA), followed by RNAeasy column purification (Qiagen). Cy3-labeled cRNA (1.5 μg) was fragmented at 60°C for 30 minutes in a reaction volume of 50 μl containing the Fragmentation Buffer and Blocking Agent included in the kit (Agilent). On completion of the fragmentation reaction, 50 μl of the HI-RPM Hybridization Buffer (Agilent) was added to the fragmentation mixture, and the samples were hybridized to Agilent SurePrint G3 Mouse GE 8 × 60 K Microarrays (G4852A, Agilent) for 17 hours at 65°C in a rotating Agilent hybridization oven. After hybridization, the microarrays were washed for 1 minute at room temperature with GE Wash Buffer 1 (Agilent) and for 1 minute with 37°C GE Wash Buffer 2 (Agilent), then dried immediately by a brief centrifugation. Immediately after being washed, the slides were scanned on a DNA Microarray Scanner (G2565CA, Agilent) using the one color scan setting for 8 × 60 K array slides. The scanned images were analyzed with the Feature Extraction Software v10.7.3.1 (Agilent) using default parameters to obtain background-subtracted and spatially detrended Processed Signal intensities.

For the clustering analysis, the normalized data were narrowed down by the cut-off values of each expression signal (>50) and fold change (>1.5, for the signal of severely compressed spinal cords versus the signal of mildly compressed spinal cords). The heat map was visualized by Gene Spring GX12 (Agilent). Pathway enrichment analysis was performed for the genes that showed differences on the microarray. RT-PCR was performed on an ABI 7900HT (Applied Biosystems, CA, USA) with TaqMan probes (Applied Biosystems).

### Histological analysis

*Twy* mice were anesthetized and transcardially perfused with 4% paraformaldehyde in 0.1 M PBS. The spinal cord and spinal canal were removed and immersed in Decalcifying Solution B (Dako, Glostrup, Denmark) for three days. These samples were then embedded in OCT compound (Sakura Finetechnical Co., Ltd., Tokyo, Japan) and sectioned in the axial plane at 20 μm on a cryostat (Leica CM3050 S, Wetzlar, Germany). The spinal cords and spinal canals were histologically evaluated by Hematoxylin-eosin (HE) staining and immunohistochemistry. The tissue sections were stained with the following primary antibodies: anti-Iba-1 (rabbit IgG, 1:200, Wako, Osaka, Japan), anti-CD86 (rat IgG, 1:200, Abcam, Cambridge, UK), anti-arginase-1 (goat IgG, 1:200, Santa Cruz Biotechnology, CA, USA), anti-Cyr61 (rabbit IgG, 1:200, Santa Cruz Biotechnology), anti-C1q (goat IgG, 1:200, Santa Cruz Biotechnology), and anti-PSD95 (mouse IgG_2a_, 1:200, Millipore, MA, USA). The samples were examined with an inverted fluorescence microscope (BZ 9000; Keyence Co., Osaka, Japan) and an LSM 700 confocal laser-scanning microscope (Carl Zeiss, Munich, Germany). To quantify the HE-stained and C1q-immunostained sections, images obtained with the BZ9000 microscope were analyzed using Keyence Analysis Software (Keyence Co.). Constant threshold values were maintained for all the analyses. HE-stained images were taken at C2/3 (the lesion epicenter) in axial sections at × 20 magnification to measure the transverse area of the spinal cord. The CD86/Iba1-, Arg-1/Iba1-, and Cyr61-stained images were automatically captured at the compressed spinal cord area in axial sections at × 200 magnification, and the CD86/Iba1- and Arg-1/Iba1-positive cells per field of view in compressed spinal cords were quantified.

### Immuno-electron microscopy

Cryosections of severely compressed *twy* mouse spinal cord (20 μm) were incubated with 5% block ace (DS Pharma Biomedical., Osaka, Japan), 0.1% Saponin in 0.1 M phosphate buffer for 1 h. Sections were immunostained with a primary rabbit anti-Iba antibody (1:100 Wako) for 72 h, and nanogold-conjugated anti-rabbit secondary antibody (1:100 Invitrogen) for 24 h at 4°C. After fixation in 2.5% glutaraldehyde, the nanogold signals were enhanced with the HQ-Silver kit (Nanoprobes Inc.) for 10 minutes. The samples were post-fixed with 0.5% osmium tetroxide, dehydrated through ethanol, acetone, and QY1, and embedded in Epon. Ultrathin (80 nm) sagittal spinal cord sections were stained with uranyl acetate and lead citrate for 10 and 12 minutes, respectively. The sections were examined under a transmission electron microscope (JEOL model 1230) and photographed using a Digital Micrograph 3.3 (Gatan Inc., CA, USA).

### Statistical analysis

All values are presented as the mean ± standard error of the mean (s.e.m.). An unpaired two-tailed Student’s *t*-test was used to determine the significance of differences in the behavioral, transcriptome, histological findings of each group. For all statistical analyses, significance was defined as *P* <0.05. GraphPad Prism software (version 5.0d) was used for the analyses (GraphPad Software, Inc., CA, USA).

## Results

**Figure 1:**
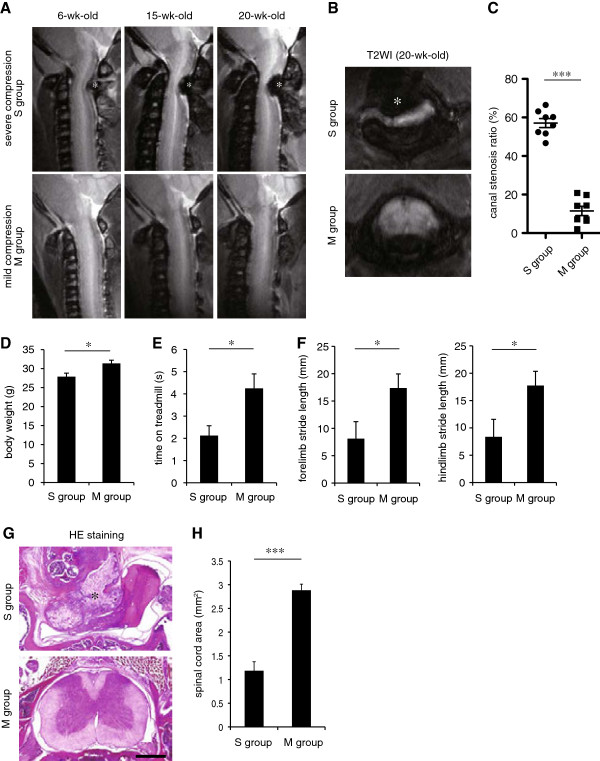
**Physical and histological differences between severely and mildly compressed spinal cord in tip-toe walking Yoshimura *(twy) *mice. (A)** Representative sagittal T2-weighted images of the severely compressed (S group) and mildly compressed (M group) spinal cords in *twy* mice (6, 15, and 20 weeks of age). **(B)** Representative axial T2-weighted images of the S and M groups at 20 weeks of age. Magnetic resonance imaging (MRI) of the S group **(A, B)** showed clear cervical spinal cord compression resulting from C2/3 ligamentous calcification (*). **(C)** The canal stenosis ratio of the S group was significantly higher than that of the M group (*n* = 8 mice per group). ***P <0.001. **(D)** Body weight in each group (*n* = 8 mice per group). *P <0.05. **(E, F)** Motor functional analyses: Latency on the rotating rod and stride length in each group (*n* = 8 mice per group). *P <0.05. **(G)** Representative HE-stained axial images of the S and M groups in *twy* mice. Scale bar: 500 μm. **(H)** Quantitative analysis of the spinal cord area in the S and M groups (*n* = 4 mice per group). ***P <0.001.

High-resolution MRI was performed when the mice were 6, 15, and 20 weeks old, as reported previously (Figure 1A) [10]. In the *twy* mice, spinal cord compression progressed at the C2/3 level due to ligamentous calcification. Twenty *twy* mice were divided into two groups according to the MRI findings: a severe compression group (*n* = 8, S group) and a mild compression group (*n* = 8, M group). The canal stenosis ratio was higher than 45% in the S group (average, 57.1%) and less than 25% in the M group (average, 11.1%) (Figure 1B and 1C). The other four *twy* mice were excluded from the analysis because they had moderate compression and could not be assigned to either group. The body weight was significantly lower in the S group than the M group (Figure 1D), and both the Rotarod treadmill latency and stride length were significantly decreased in the S group compared to the M group (Figure 1E and 1F). Consistent with the MRI findings, the spinal cord area of axial sections in the S group was significantly smaller than in the M group (Figure 1G and 1H).

**Figure 2:**
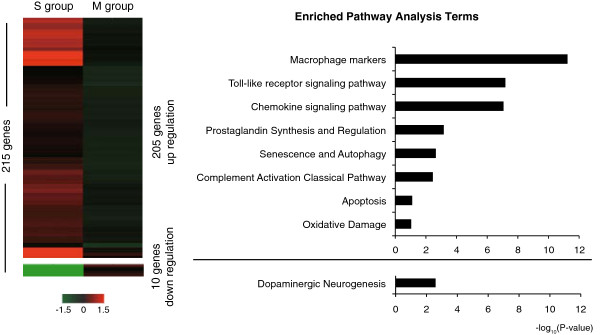
**Microarray analysis between severely and mildly compressed spinal cord in tip-toe walking Yoshimura (*twy) *mice.** Heat map (left panel) of 215 genes that differed significantly between the S and M groups (*n* = 8 mice per group). Upregulated genes in the S group are red, and downregulated genes are green. Scale is shown at the bottom. Pathway analysis terms (right panel) of the genes that differed between the S and M groups.

To investigate the pathophysiology of the compressed spinal cord in detail, microarray analysis was performed for the S and M groups (Figure 2), as previously described [20]. This analysis revealed that the expression levels of 215 genes were significantly different between the S group and the M group; 205 genes showed increased expression in the S group, and 10 showed decreased expression. Pathway analysis revealed that the genes expressed at higher levels in the S group were enriched for terms related to macrophage markers, Toll-like receptor (TLR) signaling, and chemokine signaling, which regulate inflammation and gliosis in the injured spinal cord [21]. Furthermore, genes related to prostaglandin synthesis and regulation and to oxidative damage, which suggest ischemia of the compressed spinal cord [22], were upregulated in the S group. On the other hand, Neurogenin-2, which mainly regulates the differentiation of dopaminergic neurons (‘dopaminergic neurogenesis’) [23], was downregulated in the S group. Autophagy and apoptosis pathway components were also upregulated in the S group, consistent with previous reports [11,24].

**Figure 3:**
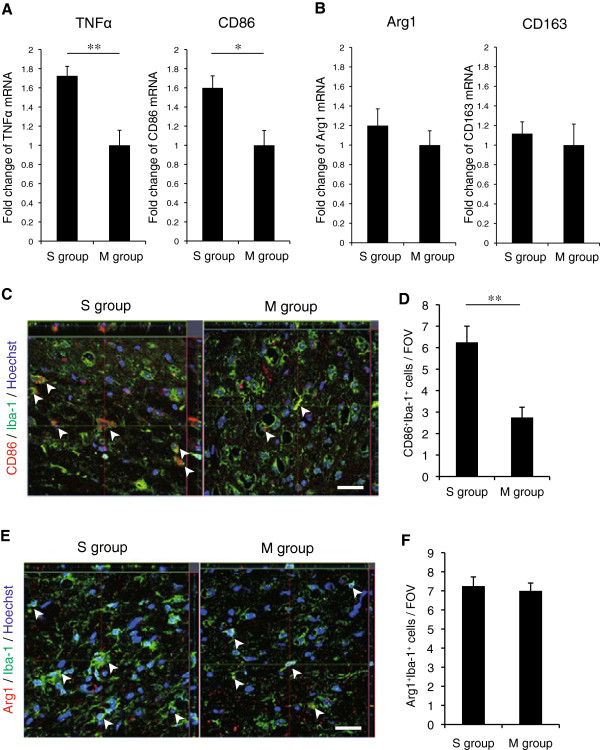
**M1 macrophages were the dominant activated macrophage phenotype in the severely compressed spinal cord. (A, B)** Fold change of the mRNAs for TNFα and CD86 (M1 macrophage markers), and for arginase1 and CD163 (M2 macrophage markers), determined by RT-PCR (*n* = 4 mice per group). **P <0.01, *P <0.05. **(C)** Representative magnified images of CD86/Iba-1 double-positive cells (arrowheads) in the S and M groups. Scale bar: 20 μm. **(D)** Quantitative analysis of the number of CD86/Iba-1 double-positive cells per field of view. **P <0.01. **(E)** Representative magnified image of arginase1 (Arg1)/Iba-1 double-positive cells in the S and M groups. Scale bar: 20 μm. **(F)** There was no significant difference in the Arg1/Iba-1 double-positive cells (arrowheads) between the S and M groups.

To evaluate the macrophage phenotype in the two groups, cervical spinal cord samples were subjected to RT-PCR and histological analyses. The mRNAs encoding TNFα and CD86, markers of the M1 phenotype, were significantly increased in the S group compared to the M group (Figure 3A), whereas there was no significant difference in the mRNAs for arginase1 or CD163, which indicate the M2 phenotype (Figure 3B). Histological analyses also showed that the number of double-positive cells for CD86 and Iba1, a microglia/macrophage marker, per field of view (FOV) (1 FOV = 100 × 100 μm^2^) was significantly higher in the S than in the M group (Figure 3C and 3D), whereas there was no significant difference in the number of Iba1/arginase1-positive cells (Figure 3E and 3F).

**Figure 4:**
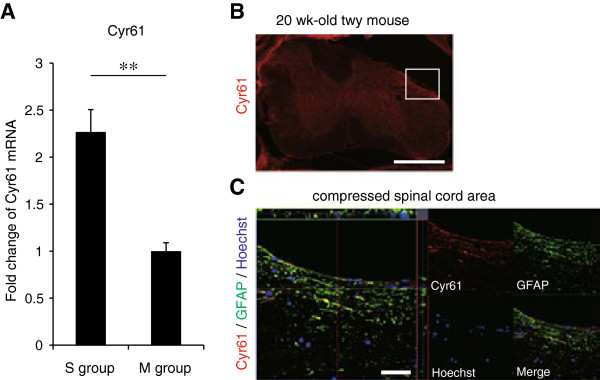
**Upregulation of Cyr61 in the severely compressed spinal cord. (A)** Fold change of the Cyr61 mRNA by RT-PCR (*n* = 4 mice per group). **P <0.01. **(B, C)** Cyr61-positive cells were located at the compressed area and highly colocalized with reactive astrocytes. Scale bar: 500 μm **(B)**, 20 μm **(C)**.

M1 macrophages are recruited by chemotaxis in response to cysteine-rich protein 61 (Cyr61) [25], which is induced by mechanical stress [26,27]. We therefore examined the gene expression of Cyr61 in the cervical spinal cord of the S and M groups. Cyr61 was significantly upregulated in the S compared to the M group (Figure 4A), and Cyr61-positive cells were located at the compressed area and colocalized extensively with reactive astrocytes (Figure 4B and 4C).

**Figure 5:**
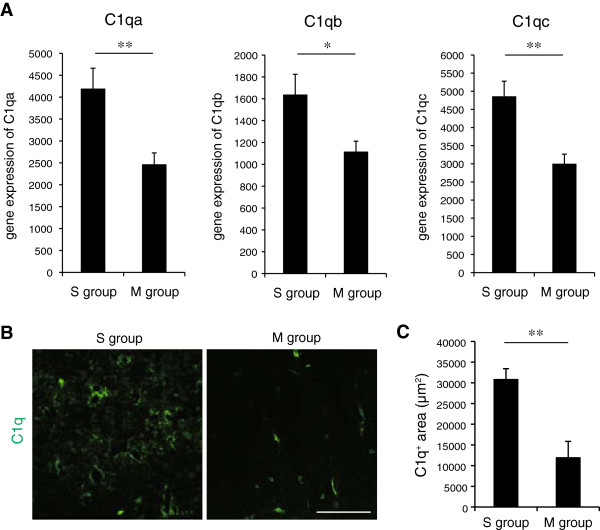
**Upregulation of the complement classical pathway in the severely compressed spinal cord. (A)** Expression of C1qa, C1qb, and C1qc mRNAs determined by microarray analysis (*n* = 8 mice per group). **P <0.01, *P <0.05. **(B)** Representative images of the C1q-positive area in the S and M groups. Scale bar: 50 μm. **(C)** Quantitative analysis of the C1q-positive area in the S and M groups (*n* = 4 mice per group). **P <0.01.

**Figure 6:**
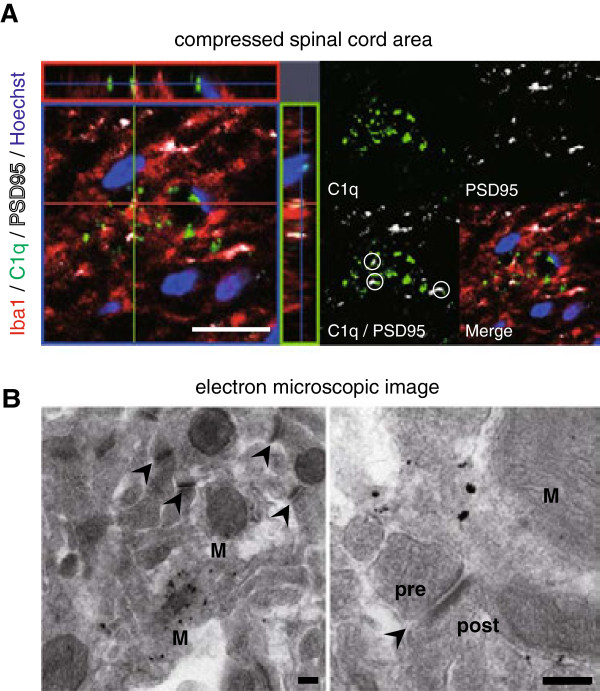
**Classical complement activation pathway-mediated synapse elimination in the severely compressed spinal cord. (A)** Representative magnified image of the Iba-1/C1q/PSD95 triple-positive area in the compressed spinal cord. Many C1q-positive puncta were colocalized with the postsynaptic protein PSD95 (several examples are circled). Scale bar: 10 μm. **(B)** Electron microscopic image of the compressed spinal cord, showing extensive contact between presynaptic/postsynaptic elements and activated microglia/macrophage processes. Scale bar: 0.2 μm. (arrowhead; synapse, pre; presynapse, post; postsynapse, M; microglia/macrophage).

To examine the mechanism of the neurodegeneration associated with inflammation in the chronically compressed spinal cord, we focused on the complement activation classical pathway (Figure 2). Previous reports suggested that at the early stage of neurodegenerative diseases and normal aging, C1q plays an important role in the pathophysiological process that leads to synapse loss and ultimately to neuronal death [28-31]. Our microarray analysis showed that the C1qa, C1qb, and C1qc expression levels were significantly higher in the S than the M group (Figure 5A). The area of punctate C1q staining was also significantly greater in the S than the M group (Figure 5B and 5C). Interestingly, many of the C1q-positive puncta that were close to microglia/macrophages in the compressed spinal cord were associated with synaptic puncta identified by double immunostaining with synaptic markers such as PSD-95 (Figure 6A). To confirm that there was direct contact between the microglia/macrophages and synaptic structures, immune-electron microscopic examination was performed. Consistent with the confocal imaging, this analysis showed that the microglia/macrophages made direct contact with both presynaptic and postsynaptic structures (Figure 6B). These observations indicated that classically activated microglia/macrophages had migrated to the compressed spinal cord and eliminated synaptic terminals.

## Discussion

In the present study, we showed that severe chronic progressive spinal cord compression in *twy* mice caused more body weight loss and neurological deficits in motor function than milder spinal cord progression. Furthermore, M1 macrophage-dominant inflammation was present in mice with a severely compressed spinal cord. In agreement, Cyr61, an inducer of M1 macrophages, was also markedly upregulated in these spinal cords. Furthermore, immunostaining and electron microscopic analyses indicated that the inflammatory C1q complement cascade eliminated synapse formation, resulting in neurodegeneration.

Macrophages are typically divided into classically activated (M1) and alternatively activated (M2) macrophages [32]. M1 macrophages, activated via TLRs, produce proinflammatory cytokines and oxidative metabolites [33]. Here we found that the M1 macrophage and TLR signals were activated in the chronically compressed spinal cord. These results were consistent with the distribution of M1 macrophages in traumatic spinal cord injury that continues even during the chronic phase [34,35]. The shift to M1 macrophages, which have deleterious and cytotoxic effects [36], may represent the main pathology of the neurodegeneration that accompanies chronic spinal cord compression.

Although the extracellular matrix has been classically viewed as an inert scaffold, recent studies have revealed that it influences diverse aspects of cellular behavior and function [37]. Cyr61 is a matricellular protein that is highly expressed at sites of inflammation, where its ability to regulate gene expression in macrophages plays an important role [25,38]. In addition, various mechanical stresses induce Cyr61 expression in cartilage/bone tissues and periodontal ligaments [26,39]. Our present data indicated that Cyr61 is significantly upregulated in the chronically, severely compressed spinal cord and colocalizes extensively with reactive astrocytes. These findings suggest that Cyr61 engages in a distinct intracellular signaling cascade in microglia/macrophages and promotes M1 macrophage recruitment in the compressed spinal cord.

Microglia/macrophages were recently identified as the phagocytes responsible for eliminating tagged synapses, via classical complement signaling [40], and the complement cascade is strongly induced in the brain tissues of patients with various neurodegenerative diseases [41]. Interestingly, in a mouse model of glaucoma, a relatively common neurodegenerative disease related to high intraocular pressure, the classical complement pathway is upregulated long before retinal ganglion cell death occurs [28]. Yet another study suggested that initiation of the classical complement pathway via C1q is detrimental to recovery after spinal cord injury [42]. The present microarray and immunohistochemical analyses showed that the classical complement pathway via C1q was significantly upregulated in the severely compressed spinal cord. Our findings raise the intriguing possibility that Clq may also be involved in synapse elimination in the chronically compressed spinal cord. Future studies should examine whether the inhibition of C1q in animal models of chronic spinal cord compression hinders the associated neurodegenerative changes.

Previous studies on the surgical outcomes of CCM patients demonstrated that the postoperative recovery was poor in those with severe canal stenosis, because irreversible changes had occurred in the spinal cord [5]. Recent studies have revealed that neural stem cell therapy can be an effective treatment for previously incurable nervous system disorders, such as spinal cord injury [43-47]. Therefore, an appropriate stem cell treatment for CCM should be examined in future studies.

To our knowledge, these data are the first to document the detailed pathophysiology of the inflammatory response in an animal model of chronic spinal cord compression. The clinical implications are noteworthy, because manipulation of the classical complement cascade in the chronically compressed spinal cord could be a strategy for minimizing synapse loss and secondary neurodegeneration due to inflammation. We believe that our findings are valuable for future research on the alterations taking place in the inflammatory environment in CCM.

## Abbreviations

CCM: cervical compressive myelopathy; Cyr61: cysteine-rich protein 61; FOV: field of view; RT-PCR: reverse transcriptase polymerase chain reaction; TLR: toll-like receptor; *twy*: tip-toe walking Yoshimura.

## Competing interests

H. Okano is the scientific consultant of San Bio, Inc; Eisai Co Ltd; and Daiichi Sankyo Co Ltd. The remaining authors declare that they have no competing interests.

## Authors’ contributions

MT, HO, and MN conceived and designed the experiments. MT, SK, YK, SS, and KH performed the experiments. MT, SK, and SS analyzed the data. YT, HO, and MN contributed the reagents, materials, and analysis tools. MT, HO, and MN wrote the paper. All authors read and approved the final manuscript.

## References

[B1] Bohlman HH Emery SE The pathophysiology of cervical spondylosis and myelopathy Spine (Phila Pa 1976) 1988 13 843 846 10.1097/00007632-198807000-00025 3057649

[B2] Fehlings MG Skaf G A review of the pathophysiology of cervical spondylotic myelopathy with insights for potential novel mechanisms drawn from traumatic spinal cord injury Spine (Phila Pa 1976) 1998 23 2730 2737 10.1097/00007632-199812150-00012 9879098

[B3] Kameyama T Hashizume Y Ando T Takahashi A Yanagi T Mizuno J Spinal cord morphology and pathology in ossification of the posterior longitudinal ligament Brain 1995 118 263 278 10.1093/brain/118.1.263 7895010

[B4] Mizuno J Nakagawa H Chang HS Hashizume Y Postmortem study of the spinal cord showing snake-eyes appearance due to damage by ossification of the posterior longitudinal ligament and kyphotic deformity Spinal Cord 2005 43 503 507 10.1038/sj.sc.3101727 15753964

[B5] Baba H Imura S Kawahara N Nagata S Tomita K Osteoplastic laminoplasty for cervical myeloradiculopathy secondary to ossification of the posterior longitudinal ligament Int Orthop 1995 19 40 45 7768658 10.1007/BF00184913

[B6] Iwasaki M Okuda S Miyauchi A Sakaura H Mukai Y Yonenobu K Yoshikawa H Surgical strategy for cervical myelopathy due to ossification of the posterior longitudinal ligament: part 1: clinical results and limitations of laminoplasty Spine (Phila Pa 1976) 2007 32 647 653 10.1097/01.brs.0000257560.91147.86 17413469

[B7] Okawa A Nakamura I Goto S Moriya H Nakamura Y Ikegawa S Mutation in Npps in a mouse model of ossification of the posterior longitudinal ligament of the spine Nat Genet 1998 19 271 273 10.1038/956 9662402

[B8] Uchida K Baba H Maezawa Y Kubota C Progressive changes in neurofilament proteins and growth-associated protein-43 immunoreactivities at the site of cervical spinal cord compression in spinal hyperostotic mice Spine (Phila Pa 1976) 2002 27 480 486 10.1097/00007632-200203010-00008 11880833

[B9] Yamaura I Yone K Nakahara S Nagamine T Baba H Uchida K Komiya S Mechanism of destructive pathologic changes in the spinal cord under chronic mechanical compression Spine (Phila Pa 1976) 2002 27 21 26 10.1097/00007632-200201010-00008 11805631

[B10] Takano M Komaki Y Hikishima K Konomi T Fujiyoshi K Tsuji O Toyama Y Okano H Nakamura M In vivo tracing of neural tracts in tiptoe walking Yoshimura mice by diffusion tensor tractography Spine (Phila Pa 1976) 2013 38 E66 E72 10.1097/BRS.0b013e31827aacc2 23124261

[B11] Yu WR Baptiste DC Liu T Odrobina E Stanisz GJ Fehlings MG Molecular mechanisms of spinal cord dysfunction and cell death in the spinal hyperostotic mouse: implications for the pathophysiology of human cervical spondylotic myelopathy Neurobiol Dis 2009 33 149 163 10.1016/j.nbd.2008.09.024 19006686

[B12] Yato Y Fujimura Y Nakamura M Watanabe M Yabe Y Decreased choline acetyltransferase activity in the murine spinal cord motoneurons under chronic mechanical compression Spinal Cord 1997 35 729 734 10.1038/sj.sc.3100529 9392042

[B13] Yu WR Liu T Kiehl TR Fehlings MG Human neuropathological and animal model evidence supporting a role for Fas-mediated apoptosis and inflammation in cervical spondylotic myelopathy Brain 2011 134 1277 1292 10.1093/brain/awr054 21490053

[B14] Uchida K Baba H Maezawa Y Furukawa S Furusawa N Imura S Histological investigation of spinal cord lesions in the spinal hyperostotic mouse (twy/twy): morphological changes in anterior horn cells and immunoreactivity to neurotropic factors J Neurol 1998 245 781 793 10.1007/s004150050287 9840350

[B15] Baltes C Radzwill N Bosshard S Marek D Rudin M Micro MRI of the mouse brain using a novel 400 MHz cryogenic quadrature RF probe NMR Biomed 2009 22 834 842 10.1002/nbm.1396 19536757

[B16] Bosshard SC Baltes C Wyss MT Mueggler T Weber B Rudin M Assessment of brain responses to innocuous and noxious electrical forepaw stimulation in mice using BOLD fMRI Pain 2010 151 655 663 10.1016/j.pain.2010.08.025 20851520

[B17] Baba H Furusawa N Fukuda M Maezawa Y Imura S Kawahara N Nakahashi K Tomita K Potential role of streptozotocin in enhancing ossification of the posterior longitudinal ligament of the cervical spine in the hereditary spinal hyperostotic mouse (twy/twy) Eur J Histochem 1997 41 191 202 9359030

[B18] Takano M Hikishima K Fujiyoshi K Shibata S Yasuda A Konomi T Hayashi A Baba H Honke K Toyama Y Okano H Nakamura M MRI characterization of paranodal junction failure and related spinal cord changes in mice PLoS One 2012 7 e52904 10.1371/journal.pone.0052904 23300814 PMC3531327

[B19] Ogura H Matsumoto M Mikoshiba K Motor discoordination in mutant mice heterozygous for the type 1 inositol 1,4,5-trisphosphate receptor Behav Brain Res 2001 122 215 219 10.1016/S0166-4328(01)00187-5 11334652

[B20] Mistry DS Chen Y Sen GL Progenitor function in self-renewing human epidermis is maintained by the exosome Cell Stem Cell 2012 11 127 135 10.1016/j.stem.2012.04.022 22770246 PMC3392610

[B21] Kigerl KA Lai W Rivest S Hart RP Satoskar AR Popovich PG Toll-like receptor (TLR)-2 and TLR-4 regulate inflammation, gliosis, and myelin sparing after spinal cord injury J Neurochem 2007 102 37 50 10.1111/j.1471-4159.2007.04524.x 17403033

[B22] Long HQ Li GS Hu Y Wen CY Xie WH HIF-1alpha/VEGF signaling pathway may play a dual role in secondary pathogenesis of cervical myelopathy Med Hypotheses 2012 79 82 84 10.1016/j.mehy.2012.04.006 22546754

[B23] Kele J Simplicio N Ferri AL Mira H Guillemot F Arenas E Ang SL Neurogenin 2 is required for the development of ventral midbrain dopaminergic neurons Development 2006 133 495 505 10.1242/dev.02223 16410412

[B24] Tanabe F Yone K Kawabata N Sakakima H Matsuda F Ishidou Y Maeda S Abematsu M Komiya S Setoguchi T Accumulation of p62 in degenerated spinal cord under chronic mechanical compression: functional analysis of p62 and autophagy in hypoxic neuronal cells Autophagy 2011 7 1462 1471 10.4161/auto.7.12.17892 22082874 PMC3288020

[B25] Bai T Chen CC Lau LF Matricellular protein CCN1 activates a proinflammatory genetic program in murine macrophages J Immunol 2010 184 3223 3232 10.4049/jimmunol.0902792 20164416 PMC2832719

[B26] Chaqour B Goppelt-Struebe M Mechanical regulation of the Cyr61/CCN1 and CTGF/CCN2 proteins FEBS J 2006 273 3639 3649 16856934 10.1111/j.1742-4658.2006.05360.x

[B27] Kivela R Kyrolainen H Selanne H Komi PV Kainulainen H Vihko V A single bout of exercise with high mechanical loading induces the expression of Cyr61/CCN1 and CTGF/CCN2 in human skeletal muscle J Appl Physiol 2007 103 1395 1401 10.1152/japplphysiol.00531.2007 17673559

[B28] Stevens B Allen NJ Vazquez LE Howell GR Christopherson KS Nouri N Micheva KD Mehalow AK Huberman AD Stafford B Sher A Litke AM Lambris JD Smith SJ John SW Barres BA The classical complement cascade mediates CNS synapse elimination Cell 2007 131 1164 1178 10.1016/j.cell.2007.10.036 18083105

[B29] Zhang Z Pinto AM Wan L Wang W Berg MG Oliva I Singh LN Dengler C Wei Z Dreyfuss G Dysregulation of synaptogenesis genes antecedes motor neuron pathology in spinal muscular atrophy Proc Natl Acad Sci U S A 2013 110 19348 19353 10.1073/pnas.1319280110 24191055 PMC3845193

[B30] Stephan AH Madison DV Mateos JM Fraser DA Lovelett EA Coutellier L Kim L Tsai HH Huang EJ Rowitch DH Berns DS Tenner AJ Shamloo M Barres BA A dramatic increase of C1q protein in the CNS during normal aging J Neurosci 2013 33 13460 13474 10.1523/JNEUROSCI.1333-13.2013 23946404 PMC3742932

[B31] Naito AT Sumida T Nomura S Liu ML Higo T Nakagawa A Okada K Sakai T Hashimoto A Hara Y Shimizu I Zhu W Toko H Katada A Akazawa H Oka T Lee JK Minamino T Nagai T Walsh K Kikuchi A Matsumoto M Botto M Shiojima I Komuro I Complement C1q activates canonical Wnt signaling and promotes aging-related phenotypes Cell 2012 149 1298 1313 10.1016/j.cell.2012.03.047 22682250 PMC3529917

[B32] Gordon S Alternative activation of macrophages Nat Rev Immunol 2003 3 23 35 10.1038/nri978 12511873

[B33] Mantovani A Sica A Sozzani S Allavena P Vecchi A Locati M The chemokine system in diverse forms of macrophage activation and polarization Trends Immunol 2004 25 677 686 10.1016/j.it.2004.09.015 15530839

[B34] Kigerl KA Gensel JC Ankeny DP Alexander JK Donnelly DJ Popovich PG Identification of two distinct macrophage subsets with divergent effects causing either neurotoxicity or regeneration in the injured mouse spinal cord J Neurosci 2009 29 13435 13444 10.1523/JNEUROSCI.3257-09.2009 19864556 PMC2788152

[B35] Guerrero AR Uchida K Nakajima H Watanabe S Nakamura M Johnson WE Baba H Blockade of interleukin-6 signaling inhibits the classic pathway and promotes an alternative pathway of macrophage activation after spinal cord injury in mice J Neuroinflammation 2012 9 40 10.1186/1742-2094-9-40 22369693 PMC3310810

[B36] Laskin DL Macrophages and inflammatory mediators in chemical toxicity: a battle of forces Chem Res Toxicol 2009 22 1376 1385 10.1021/tx900086v 19645497 PMC2787782

[B37] Aszodi A Legate KR Nakchbandi I Fassler R What mouse mutants teach us about extracellular matrix function Annu Rev Cell Dev Biol 2006 22 591 621 10.1146/annurev.cellbio.22.010305.104258 16824013

[B38] Jun JI Lau LF The matricellular protein CCN1 induces fibroblast senescence and restricts fibrosis in cutaneous wound healing Nat Cell Biol 2010 12 676 685 10.1038/ncb2070 20526329 PMC2919364

[B39] Li Y Li M Tan L Huang S Zhao L Tang T Liu J Zhao Z Analysis of time-course gene expression profiles of a periodontal ligament tissue model under compression Arch Oral Biol 2012 58 511 522 23116693 10.1016/j.archoralbio.2012.10.006

[B40] Aguzzi A Barres BA Bennett ML Microglia: scapegoat, saboteur, or something else? Science 2013 339 156 161 10.1126/science.1227901 23307732 PMC4431634

[B41] Stephan AH Barres BA Stevens B The complement system: an unexpected role in synaptic pruning during development and disease Annu Rev Neurosci 2012 35 369 389 10.1146/annurev-neuro-061010-113810 22715882

[B42] Galvan MD Luchetti S Burgos AM Nguyen HX Hooshmand MJ Hamers FP Anderson AJ Deficiency in complement C1q improves histological and functional locomotor outcome after spinal cord injury J Neurosci 2008 28 13876 13888 10.1523/JNEUROSCI.2823-08.2008 19091977 PMC2680920

[B43] Amoh Y Li L Campillo R Kawahara K Katsuoka K Penman S Hoffman RM Implanted hair follicle stem cells form Schwann cells that support repair of severed peripheral nerves Proc Natl Acad Sci U S A 2005 102 17734 17738 10.1073/pnas.0508440102 16314569 PMC1308908

[B44] Amoh Y Li L Katsuoka K Hoffman RM Multipotent hair follicle stem cells promote repair of spinal cord injury and recovery of walking function Cell Cycle 1865–1869 2008 7 10.4161/cc.7.12.6056 18583926

[B45] Amoh Y Li L Katsuoka K Penman S Hoffman RM Multipotent nestin-positive, keratin-negative hair-follicle bulge stem cells can form neurons Proc Natl Acad Sci U S A 2005 102 5530 5534 10.1073/pnas.0501263102 15802470 PMC556262

[B46] Li L Mignone J Yang M Matic M Penman S Enikolopov G Hoffman RM Nestin expression in hair follicle sheath progenitor cells Proc Natl Acad Sci U S A 2003 100 9958 9961 10.1073/pnas.1733025100 12904579 PMC187900

[B47] Liu F Uchugonova A Kimura H Zhang C Zhao M Zhang L Koenig K Duong J Aki R Saito N Mii S Amoh Y Katsuoka K Hoffman RM The bulge area is the major hair follicle source of nestin-expressing pluripotent stem cells which can repair the spinal cord compared to the dermal papilla Cell Cycle 2011 10 830 839 10.4161/cc.10.5.14969 21330787

